# Medical Books

**Published:** 1872-11

**Authors:** 


MEDICAL BOOKS.—We are in receipt of several catalogues of medical works from the publishers. The one issued by Messrs. Wm. Wood & Co. is perhaps the most elaborate, being in colored paper covers with illustrations. Among the works in preparation by this house, we notice the following: Lectures on the Palsies and Kindred Disorders of the Nervous System, by Meredith Clymer, M. D.; Clinical Lectures on Diseases of Genito-Urinary Organs and their Treatment, by J. W. S. Gouley, M. D.; and a Treatise on the Diseases of the Ear, by D. B. St. John Rosa, M. D. The works which are issued by this firm are printed in clear type and finely and strongly bound. Messrs. Wood & Co.'s address is 27 Great Jones Street, New York.

Mr. Henry C. Lea, so long and favorably known as a publisher of medical books presents a catalogue as usual full of standard works. He announces as shortly to be published Erichsen's Science and Art of Surgery. Sixth Edition In Two Volumes. Also a work on the Treatment of Skin Diseases, by McCall Anderson, M. D.; and A Manunl of Surgery, by Thomas Bryant, F. R. C. S.

Messrs. Lindsay & Blakiston are busy getting ready for publication several new works. They announce: The Second London Edition of Sœlberg Wells' Treatises on Diseases of the Eye, Trousseau's Clinical Medicine, complete in two volumes, and Biddle's Materia Medica, fifth edition, besides several other works in active preparation.

D. Appleton & Co., of New York, have recently published some very excellent medical works, of which we have had occasion from time to time to speak. They have several new works in press, among others, Wellson on Diseases of the Ovaries, and Barker on Puerperal Diseases. They announce, also, a work on the Nervous System, by Austin Flint, Jr., M. D., and Wm. A. Hammond, M. D. consisting of the fourth volume of Flint's Physiology. "The Nervous System" and Hammond's Treatise of Diseases of the Nervous System, in two volumes.

